# Green Extraction of Polyphenols from Waste Bentonite to Produce Functional Antioxidant Excipients for Cosmetic and Pharmaceutical Purposes: A Waste-to-Market Approach

**DOI:** 10.3390/antiox11122493

**Published:** 2022-12-19

**Authors:** Giulia Di Prima, Elena Belfiore, Martina Migliore, Amalia Giulia Scarpaci, Giuseppe Angellotti, Ignazio Restivo, Mario Allegra, Vincenzo Arizza, Viviana De Caro

**Affiliations:** 1 Department of Biological, Chemical and Pharmaceutical Sciences and Technologies, University of Palermo, 90123 Palermo, Italy; 2 Department of Surgical, Oncological and Oral Sciences, University of Palermo, 90127 Palermo, Italy; 3 Centro Interdipartimentale di Ricerca Riutilizzo Bio-Based Degli Scarti da Matrici Agroalimentari (RIVIVE), University of Palermo, 90128 Palermo, Italy

**Keywords:** bentonite, waste product, extraction, polyphenols, grape processing industry, propylene glycol, PEG200, cosmeceutical excipients, pharmaceutical excipients, waste-to-market approach

## Abstract

In an ever-growing perspective of circular economy, the development of conscious, sustainable and environmental-friendly strategies to recycle the waste products is the key point. The scope of this work was to validate the waste bentonite from the grape processing industries as a precious matrix to extract polyphenols by applying a waste-to-market approach aimed at producing novel functional excipients. The waste bentonite was recovered after the fining process and opportunely pre-treated. Subsequently, both the freeze dried and the so-called “wet” bentonites were subjected to maceration. PEG200 and Propylene Glycol were selected as solvents due to their ability to dissolve polyphenols and their wide use in the cosmetic/pharmaceutical field. The extracts were evaluated in terms of yield, density, pH after water-dilution, total phenolic (Folin–Ciocalteu) and protein (Bradford) contents, antioxidant power (DPPH), amount of some representative polyphenols (HPLC-DAD), cytocompatibility and stability. Both solvents validated the bentonite as a valuable source of polyphenols and led to colored fluids characterized by an acidic pH after water-dilution. The best extract was obtained from the wet bentonite with PEG200 and highlighted the highest phenolic content and consequently the strongest antioxidant activity. Additionally, it displayed proliferative properties and resulted almost stable over time. Hence, it might be directly used as polyphenols-enriched functional novel raw material for cosmetic and pharmaceutics purposes.

## 1. Introduction

The wine industry represents one of the most important economy sectors worldwide with 35.9 million tons of grapes pressed to produce wine [1]. Italy is one of the most representative countries in this field, and Sicily in particular can be considered the cradle of several autochthonous and international vines, which are a significant and rich regional heritage. Moreover, Sicily is the Italian region with by far the largest area cultivated with organic grapes [2]. The constant spreading of grape processing and winemaking industries in Sicily contributes to the enhancement of the territorial resources, which is particularly relevant to contrast the worldwide economic crisis experienced in the last years. In an ever-growing perspective of circular economy, nowadays, great importance is given to the waste products, to their fate in particular, and to novel smart strategies capable of reducing the environmental footprint [3,4]. Among the various products generally considered waste, those obtained by the grape processing and winemaking industries have recently attracted the attention of both companies and researchers due to their potentially high content of bioactive molecules, especially polyphenols. The latter are naturally occurring functional compounds recovered in different fruits and vegetables such as citrus, orange, lemon, grapefruit, olive, pomegranate, peach and grape [5,6,7,8,9]. Plants naturally produce polyphenols as secondary metabolites useful to protect and defend themselves against environmental aggressions and pathogens (e.g., bacteria and fungi) [10]. Similarly, when properly used, polyphenols may exert beneficial and protective activities on human health due to their wide range of therapeutic properties such as antioxidant, antiaging, anti-hyperpigmentation, photoprotective, anticancer and immunomodulatory effects. These lead us to consider them precious active ingredients in pharmaceutical, food and cosmetic products [11,12,13,14,15,16,17,18,19,20,21,22]. Polyphenols are generally characterized by at least one benzene ring with one or more hydroxyl substituents [23]. They can be divided into different groups based on their chemical structure: phenolic acids, flavonoids, tannins, lignans and neolignanes, stilbenes, coumarins, curcuminoids and phenyl ethanol derivatives [24,25,26]. The main parts that are considered grape processing waste products as a source of functional polyphenols are generally vine stems, grape pomace and wine lees, which are usually recovered and subjected to (i) optional pre-treatment, (ii) extraction process, (iii) separation and concentration and (iv) final use or dehydration and storage [4]. Instead, the waste bentonite could be a novel possible source of polyphenols. The latter is a common additive used to clarify and fine musts and wines. It is a phyllosilicate belonging to the montmorillonite group, which is great for removing protein haze; however, it can also entrap such interesting molecules as polyphenols. The bentonites can be classified into natural calcium bentonite, natural sodium bentonite and activated calcium bentonite depending on the nature of the exchangeable cations, the swelling index and the native pH [27,28,29]. The bentonite is one of the most used clarifying agents in the enological field as it is naturally abundant, low cost and easy to remove from the fined product by sedimentation. However, it also displays some relevant limitations as the retention of a certain amount of must/wine and the impossibility of its reconstitution and, consequently, reuse as a clarifying agent [30]. Although only considered an abundant waste till now, bentonite may still be a valuable source of powerful polyphenols and could be further used and valorized by means of the phenolic compounds’ extraction. The effectiveness of the extraction process relies on the matrices used, the experimental conditions, the type of phenolic compounds to be extracted, the employed solvent and the applied method [31]. Based on these considerations, this work aims to develop a conscious, sustainable and environmentally-friendly strategy to give new life to the waste bentonite. In view of a waste-to-market approach, Propylene Glycol and PEG200 are here proposed as green and innovative extraction solvents as they are well-known excipients often used for pharmaceutical and cosmetic applications. Ideally, it should be possible to produce novel enriched raw materials useful in the pharmaceutic and cosmetic fields to benefit from the presence of the functional polyphenols for several purposes. Thus, the scope of this paper is to employ the waste bentonite as a starting material to produce novel excipients enriched in functional polyphenols and characterized in terms of antioxidant power, phenolic amount, protein content, cytotoxicity and stability.

## 2. Materials and Methods

### 2.1. Materials

Enobent^®^ Standard (sodium-activated bentonite in powder form) was supplied by Bono & Ditta S.p.A. (Campobello di Mazara, Trapani, Italy), both the novel (as marketed) and the waste one (after its complete use for wine clarification: 100 g of bentonite per must hL from white berried grapes). Propylene glycol and resveratrol (RSV) were purchased from A.C.E.F. Spa (Fiorenzuola D’Arda, Italy). Quercetin (QRC) was supplied from Farmalabor (Canosa di Puglia, Italy). Gallic Acid (GA), Bradford and Folin–Ciocalteu reagents were purchased by Merck (Darmstadt, Germany). Bovine serum albumin (BSA), Polyethyleneglycol 200 (PEG200) and 2,2-diphenyl-1-picrylhydrazyl free radical (DPPH) were obtained from Carlo Erba (Milan, Italy). All other chemicals and solvents (analytical grade) were purchased from Carlo Erba and were used without further purification.

### 2.2. Waste Bentonite Recovery and Storage

The waste bentonite was supplied by Bono&Ditta S.p.A. (Campobello di Mazzara, Trapani, Italy). This is intended as a discarded product, which appears as a compact, grey and humid mass, as it comes from a pressing procedure aimed at “squeezing out” all the must inside. After its complete use, the bentonite was taken from the filtration silo and stored at −20 °C. A representative sample (≈10 kg) of bentonite identifiable as belonging to the same lot (used to clarify the same starting must from white berried grapes), was then transferred into refrigerated boxes and transported to the laboratory.

### 2.3. Pre-Treatment of Waste Bentonite

The freshly obtained frozen waste bentonite was immediately pulverized in mortar, sieved, fractionated into smaller aliquots (≈100 g), sealed in polypropylene bags and stored at −80 °C (Thermo Forma ultra-freezer −86 °C mod. 902 Thermo Scientific, Waltham, MA, USA).

### 2.4. Freeze Drying and Evaluation of the Water Content

Accurately weighted amounts of bentonite were freeze dried in order to eliminate any residual water as well as to evaluate the weight water content percentage (*W_c_*%) as follows:

$$W_{c}\%=\frac{starting\;weight\;\left(g\right)-freeze\;dried\;weight\;\left(g\right)}{starting\;weight\;\left(g\right)}\;\times 100$$

The evaluation was carried out on twelve samples (*n* = 12) and results are reported as means ± Standard Error (SE).

### 2.5. Extracts Preparation

A carefully weighted amount of bentonite (1 g of freeze-dried bentonite; 2 g of untreated bentonite also named wet bentonite due to the significant water content) was mixed with 8 g of propylene glycol or PEG200 and kept at 25.0 ± 0.5 °C under vigorous magnetic stirring (Heidolph MR3001K Hotplate Stirrer with Heidolph EXT3001 Temperature Probe, Heidolph Instruments, Schwabach, Germany) for 1 h in the dark. At the end of the extraction procedure each sample was transferred into a 15-mL plastic flacon and centrifuged (12,000 rpm, 10 °C, 20 min). Furthermore, the obtained colored supernatant was filtered through a 0.45 µm nylon syringe filter and measured in terms of final weight. In Table 1 sample formula codes are reported. The bentonite:solvent ratio was chosen according to the previously obtained *W_c_*% data in order to standardize the procedure and obtain easily comparable samples.

Each extraction procedure was repeated in triplicate (*n* = 3). The yield of the process expressed as percent was calculated as follows:

$$Yield\;\%=\frac{amount\;of\;recovered\;extract\;\left(g\right)}{starting\;amount\;of\;solvent\;\left(g\right)}\times 100$$

Results are reported as means ± SE. The prepared extracts were then stored at 4 °C in the dark until subsequent analyses.

### 2.6. Density Evaluation

The density of each extract was measured by accurately weighting a fixed volume (500 µL) of each sample using an analytical balance (Mettler, Columbus, OH, USA, Mod. AE 240) and subsequently mathematically calculating the density value. Each evaluation was performed in triplicate on each extract (*n* = 9), and results are expressed as means ± SE.

### 2.7. pH Evaluation after Water Dilution

The sample solutions (100 mg mL^−1)^ were prepared by dissolving 500 mg of extract into a 5 mL volumetric amber flask using ultrapure water as a solvent. The pH of the resulting solutions was measured using a pH meter HI 2211 pH/ORP Meter, Hanna Instrument (Woonsocket, RI, USA). Each experiment was performed in triplicate on each extract (*n* = 9). Results are reported as means ± SE.

### 2.8. Evaluation of the Antioxidant Power with a DPPH Assay

Blank and sample solutions to be subjected to the DPPH assay were prepared by diluting 100 µL of fresh extraction solvents (propylene glycol or PEG200) or the obtained extracts respectively with methanol to a final volume of 5 mL (samples concentration: 20 µL mL^−1^). Then, 100 µL of the latter solutions were added to 2 mL of DPPH stock solution (40 µg mL^−1^) previously loaded into a quartz cuvette, well-mixed and immediately subjected to UV-Vis measurements using a Shimadzu 1700 instrument (Kyoto, Japan) with the appropriate calibration curve and blank. The DPPH reduction over time was monitored at room temperature by analyzing each sample every 5 min until 1 h. To accurately quantify the residual DPPH, the appropriate calibration curve was constructed by preparing six DPPH standard solutions in methanol: λ_max_ = 515 nm; linearity range: 4–40 µg mL^−1^, regression equation: Abs = 0.018 + 28.59 × [mg mL^−1^], (R = 0.999). Each experiment was performed in triplicate on each extract (*n* = 9). Results are expressed as percentage of residual DPPH ± SE as a function of incubation time.

Moreover, standard DPPH curves were obtained by analyzing gallic acid (GA) standard solutions (15–50 μg/mL) in ultrapure water according to the operative method used for the samples. The residual DPPH % value at 3 selected time points (10, 30 and 60 min) was used to construct 3 calibration curves that were helpful to compare the obtained extract to a phenolic standard in terms both of antioxidant power and rate of DPPH consumption, as already reported [32]. The obtained curves were the following: time point 10 min—regression equation: residual DPPH % = 90.65 − 1153.55 × [mg mL^−1^], (R = 0.996); time point 30 min—regression equation: residual DPPH % = 91.38 − 1293.95 × [mg mL^−1^], (R = 0.999); time point 60 min—regression equation: residual DPPH % = 90.65 − 1338.35 × [mg mL^−1^], (R = 0.999). The experiments aimed at obtaining the GA calibration curves were performed in triplicate (*n* = 3), and the results are reported as the mean of the equivalent mg of gallic acid per 1 g of extract ± SE for each time point (*n* = 9).

### 2.9. Quantitative Evaluation of Some Representative Polyphenols with HPLC-DAD

Aliquots of 200 µL of each prepared extract, which were previously filtered through a 0.22 µm PTFE siring filter, were properly diluted with methanol (1:1 *v*/*v*) and subjected to quantitative analysis using a HPLC Agilent 1260 Infinity Instrument equipped with a Quaternary Pump G1311B, a Diode Array Detector 1260 Infinity II, an automatic vialsampler G7129C and a computer integrating apparatus (OpenLAB CDS ChemStationWorkstation, Stockholm, Sweden) (injected volume: 20 µL; column temperature: 25 °C). Chromatographic separation was achieved on a reversed-phase column, Ace^®^ Excel Super C18 (5 U, 100 A, size 125 × 4.60 mm). To quantify gallic acid, resveratrol and quercetin a mobile phase consist of 0.1% (*v*/*v*) TFA solution in water (solvent A) and acetonitrile (solvent B) was used, and the following time program was set: 0–2 min isocratic conditions A:B = 90:10; gradient from 2 min A:B = 90:10 to 22 min A:B = 5:95; 22–23 min isocratic conditions A:B = 5:95, gradient from 23 min A:B = 5:95 to 25 min A:B = 90:10 and finally 25–27 min isocratic conditions A:B = 90:10. The flow rate was set at 1 mL/min, and the DAD investigation covers the range 200–800 nm. In these conditions, the retention time of gallic acid (GA), resveratrol (RSV) and quercetin (QRC) was 2.77, 11.25 and 12 min, respectively. Appropriate calibration curves for each compound were constructed. For GA, the λ_max_: 271 nm, linearity range: 0.5–100 µg/mL, regression equation: Area = −4.38 + 32,113.53 × [mg mL^−1^], (R = 0.999); the RSV: λ_max_: 305 nm, linearity range: 0.1–50 µg/mL, regression equation: Area = 49.47 + 142283.71 × [mg mL^−1^], (R = 0.999) and the QRC: λ_max_: 370 nm, linearity range: 1–100 µg/mL, regression equation: Area = −14.63 + 77,606.83 × [mg mL^−1^], (R = 0.999). Each quantitative evaluation was repeated in triplicate on each extract (*n* = 9). Results are expressed as means ± SE.

### 2.10. Determination of the Total Phenolic Content with a Folin–Ciocalteu Assay

The Folin–Ciocalteu reaction was used to quantify the total phenolic content as previously reported [33]. Briefly, sample solutions (100 mg mL^−1^) were prepared by diluting 500 mg of extract or fresh solvents into a 5 mL volumetric amber flask using ultrapure water as a solvent. Then, 50 µL of each clear solution were added to 2 mL of ultrapure water previously loaded into a 15-mL plastic tube. Subsequently, 130 µL of Folin–Ciocalteu reagent were added to the tube, mixed well and left to settle for 5 min in the dark. Finally, 370 µL of Na_2_CO_3_ solution in ultrapure water (0.2 g/mL) were added to each tube, mixed well and maintained at room temperature in the dark for 2 h. Afterwards, samples were subjected to UV-Vis measurements using a Shimadzu 1700 instrument (Kyoto, Japan) with the appropriate calibration curve and blank. Similarly, six gallic acid standard solutions in ultrapure water (50–500 µg/mL) were prepared and analyzed to construct the calibration curve as follows: λ_max_ = 760 nm; linearity range: 0.98–9.80 µg/mL, regression equation: Abs = 0.039 + 65.08 × [mg mL^−1^], (R = 0.999). Each experiment was performed in triplicate on each extract (*n* = 9). Results are expressed as equivalent mg of gallic acid per 1 g of extract ± SE.

### 2.11. Determination of the Total Protein Content with a Bradford Assay

Sample solutions (100 mg mL^−1^) were prepared by dissolving 500 mg of extract into a 5 mL volumetric amber flask using ultrapure water as a solvent. Then, 200 µL of each clear solution were added to 600 µL of ultrapure water previously loaded into a 2-mL plastic eppendorf. Subsequently, 200 µL of a Bradford reagent (B6916—Sigma Aldrich, St. Louis, MO, USA) were added, mixed well and maintained at room temperature in the dark for 30 min. Afterwards, samples were subjected to UV-Vis measurements using a Shimadzu 1700 instrument (Kyoto, Japan) with the appropriate calibration curve and blank. Similarly, five BSA standard solutions in ultrapure water were prepared and analyzed to construct the calibration curve as follows: λ_max_ = 595 nm; linearity range: 2–7 µg/mL, regression equation: Abs = 0.1700 + 0.0332 × [µg/mL], (R = 0.998). Each experiment was performed in triplicate on each extract (*n* = 9). Results are expressed as equivalent mg of BSA per 1 g of extract ± SE.

### 2.12. Cell Viability Assay

Unless stated otherwise, all reagents, including BALB/3-T3 cells, were from Merck (Milan, Italy) and of the highest purity grade commercially available. BALB/3-T3 cells were grown in DMEM supplemented with L-glutamine (2 mM), 10% fetal bovine serum (FBS), penicillin (100 U/mL) and streptomycin (100 µg/mL). Cells were maintained in the log phase by seeding them twice a week at a density of 3 × 10^5^ cells/mL in humidified 5% CO_2_ atmosphere at 37 °C (Thermo Fisher Scientific, Waltham, Massachusetts, US, Mod: 3543). The cytotoxicity of both the extracts and the fresh extraction solvents was assessed with an MTT assay as previously reported [34]. Briefly, cells at the passage that did not exceed the number 20 were seeded in a 96-well plate (Corning™, New York, NY, USA) at a density of 1.8 × 10^4^ cells/well, incubated overnight and then treated in the absence (control) or in the presence of the samples at various concentrations (2.5–20 μL/mL) for 24 h. Afterwards, the medium was carefully removed, each well was washed up with 200 μL of PBS pH 7.4 and then 200 μL of fresh medium containing MTT (5 mg mL^−1^) were added. Plates were incubated for an additional 2 h at 37 °C and then centrifuged at 1000 rpm for 5 min (Allegra X12, Beckman Coulter, Life Sciences, Milan, Italy). The supernatant was discharged and replaced with 100 µL of DMSO in order to solubilize the previously precipitated formazan crystals. The absorbance at 570 nm was measured with a LTEK A-302 plate reader (INNO, Seongnam, Republic of Korea), and the value of control cells was taken as 100% of viability. Each experiment was repeated three times in triplicate (*n* = 9), and results are expressed as means ± SE.

### 2.13. Stability Studies

The prepared extracts were stored in the dark at 4 °C for six months and then subjected to the HPLC-DAD, DPPH, Folin–Ciocalteu and Bradford analyses as described above in order to assess their stability at the employed storage conditions. Each stability assay was performed in duplicate on each extract (*n* = 6), and results are expressed as means ± SE.

### 2.14. Control Group

All the experiments and the evaluations reported above were also conducted using fresh bentonite Enobent^®^ Standard (as marketed). The results (*n* = 3) are not reported as no compounds were extracted from the control bentonite and thus the control samples (denominated G and P) did not display any antioxidant power, neither HPLC-DAD peaks, phenolic and protein contents. Additionally, no interferences on the assays carried out were detected.

### 2.15. Data Analysis

The data was expressed as mean ± standard error (SE) or standard deviation (SD). All differences were statistically evaluated with the Student’s t test or the one-way analysis of variance (ANOVA or F-test) with the minimum levels of significance with *p* < 0.05.

## 3. Results and Discussion

### 3.1. Waste Recovery, Extracts Preparation and Preliminary Evaluations

The agri-food sector produces a significant amount of waste that, in an ever-growing perspective of circular economy, can be reused as secondary raw materials (SRMs) for various purposes. In particular, waste bentonite from the grape processing and winemaking industries deserves special attention as it could represent a valuable and precious source of functional polyphenols. The bentonite is commonly used to clarify and fine musts and wine due to its ability to retain proteins which are the main leading cause of wine instability and cloudiness [35,36].

In this study, the waste bentonite originates exclusively from the processing of organic white grapes. The recovered waste bentonite appeared as a compact, grey and humid mass coming from a pressing procedure. The latter was immediately stored at −20 °C to stop or at least slow down the degradation processes of the here-contained functional substances. To the aim of producing samples as homogeneous as possible, the so-obtained bentonite was subjected to the following pre-treatments; (i) the frozen mass was pulverized in mortar to obtain a finer powder, thus enhancing the exchange surface between the bentonite and the extraction solvents. This step must be performed on the frozen bentonite as the thawed one is moist and characterized by a pasty consistency. (ii) The obtained powder was coarse sieved to remove residues such as pomace and grape seeds as well as residual big aggregates; (iii) the sieved powders were mixed and then fractionated into aliquots and then stored at −80 °C. Furthermore, carefully weighted amounts of bentonite were subjected to freeze drying. This process was conducted with the consideration that one of the main disadvantages when using the bentonite as a fining agent is related to a significant must/wine retention (3–10% of the total volume). As musts and wine are basically hydroalcoholic solutions (~78% water) containing a wide variety of chemical components, including aldehydes, esters, ketones, lipids, minerals, organic acids, phenolics, soluble proteins, sugars, vitamins, isoprenoids, lipids, and waxes [21,37], the evaluation of both the dry weight of the waste bentonite and the water content is crucial to assess sample uniformity. As expected, the calculated Water Content % (*W_c_*%) was significantly high: 54.15 ± 1.56% (*n* = 12). This result indicates that the prepared samples were uniform in terms of *W_c_*% as the observed standard error is quite narrow. Additionally, it clearly reveals that when weighing an aliquot of bentonite, just over half of its weight is actually made up of residual water. Due to these relevant results and in order to evaluate whether the freeze-drying process must be considered a required pre-treatment step, the subsequent experimental phases were conducted on both the freeze-dried bentonite and the so-called wet bentonite.

After the pre-treatment process, the extraction process parameters were accurately evaluated. In particular, the extraction method, the employed solvents and extraction duration required were the main investigated parameters. The literature reports many extraction methods frequently used to recover phenolic compounds from grape processing waste (mainly pomace and seeds), such as ultrasound [38], supercritical fluid [39], enzyme-assisted extraction [40], percolation [41] and maceration [42]. The latter consist of a traditional solid liquid extraction, which remains the most commonly used, accessible and easily scalable technique and was thus selected for the present study.

The driving force leading the choice of the solvents to be employed lies in the purpose of a waste-to-market approach. To this scope, it was necessary to identify well-known, currently used and safe liquid excipients already approved for pharmaceuticals and cosmetics and capable of acting as great solvents for polyphenols. Considering these issues, the following liquid excipients were selected:

- PEG200: it is a synthetic, highly water soluble, inert polymer widely used in cosmetics, pharmaceuticals and other consumer care products. It could be considered a hydrophilic, stable, non-toxic solvent that also displays humectants properties. Additionally, it was extensively reported to possess good solvent properties towards polyphenols. Particularly, PEG200 was selected after preliminary studies, which displayed that the use or PEGs with higher molecular weight (e.g., PEG400 and PEG600) reduces the solvent power towards polyphenols and, consequently, the efficiency of the extraction process [11,43,44].
- Propylene Glycol: it is a hydrophilic polar solvent characterized by humectants and antimicrobial activities. It is frequently employed in the cosmetic field as a moisturizer and skin conditioner due to its hygroscopic nature playing a role for stratum corneum hydration [45]. Moreover, it is also well-known in the pharmaceutic field due to its plasticizer and penetration enhancer properties. Additionally, it was already reported to possess good solvent properties towards polyphenols [11,14,46,47].

Moreover, it should be highlighted that the selected solvents led to eco-friendly and green extractions with respect to the traditional use of organic solvents.

Considering the previously observed high *W_c_*% value, the extractions were carried out on both wet and freeze-dried bentonite in order to evaluate whether the amount of water in the wet samples could have any detectable effect on the performance and yield of the process. To standardize the extraction procedure, the bentonite:solvent ratio was decided according to the water content. Particularly, 2 g of wet bentonite were soaked with 8 g of each solvent (wet bentonite:solvent = 1:4) as this volume allowed the complete wettability of the chosen bentonite amount while also permitting the obtainment of an easily mixable suspension by magnetic stirring. Consequently, as 2 g of wet bentonite comprises ≥50% (*w*/*w*) of residual water, the freeze-dried bentonite:solvent ratio was set at 1:8. The use of lower solvent amounts made the stirring procedure difficult to allow sedimentation over time, thus leading to the blocking of the magnet. On the other hand, the use of a higher amount of solvents led to too diluted samples. As a consequence, the employed ratio was the optimized one to merge the necessity of both stirring during the whole extraction process as well as producing concentrated extracts as high as possible (also because the chosen solvent could not be concentrated by evaporation).

The further modifiable extraction parameter was related to the time of maceration. The optimized extracts were obtained after 1 h of maceration. Indeed, by shorting the extraction process (e.g., 30 min), the contact time was insufficient to allow solvent penetration into the matrix and to mix the two phases well, thus leading to non-reproducible results. On the other hand, prolonged times did not affect the quality of the resulting solutions in terms of the amount of valuable polyphenols as well as antioxidant power.

Table 2 highlights the main starting outcomes when evaluating the four obtained extracts. It is noticeable that the *Yield* % values ranged between 60–80%. The extract loss is probably related to the solvents density, which led to the need of a two-step separation procedure: centrifugation and then filtration. By observing the centrifuged samples, it was noted that the solvents entrapped in the residual bentonite were not completely removable; the latter did not vary by increasing the applied centrifugal force, and this observation was completely independent from the initial presence of water (wet or freeze-dried samples). Moreover, the measured density of the extracts showed only slight increments when compared to the related fresh extraction solvent, thus indicating that the amount of water in the waste bentonite is irrelevant to the density of the final product when using the above ratios. Finally, the pH evaluation after the water dilution of accurately weighted samples of both propylene glycol, PEG200 and the four prepared extracts gave some relevant findings, preliminarily indicating the plausible effectiveness of the extraction process and thus the presence of polyphenols in the solution. Indeed, propylene glycol and PEG200 water solutions showed pH values of ≈6.5 (similar to those registered for the control extraction samples), whereas the water dilution of the four obtained extracts resulted in acidic pH values (≈4). The latter undoubtedly indicates the actual extraction of acidic compounds. These might be both organic acids, widely present in musts and wines as well as polyphenols. Indeed, it is well-known that despite their hydroxyl groups, phenols are more acidic than alcohols. This phenomenon is due to the resonance that disperses the electric charge throughout the aromatic ring(s) linked to the hydroxyl groups. Undoubtedly, the registered difference in term of pH indicated that the maceration successfully extracted from the waste bentonite such compounds responsible for the acidic pH of the diluted extracts.

To evaluate the goodness of the proposed green extraction processes and solvents, reference extraction procedures using acetone or methanol as conventional solvents were carried out. To compare the green and the conventional extracts, clarity and stability at different storage temperatures and quantification of QRC and RSV with HPLC-DAD were evaluated as parameters. However, both acetone and methanol extracts manifested some significant instability as the obtained clear solutions became turbid in a few hours, leading to precipitate formation over time even after 24 h (both when stored at room temperature or at 4 °C to minimize evaporation). This could be attributable to the ability of the organic solvents to extract other components (e.g., high amounts of proteins, polysaccharides and fibers), which were not completely soluble. However, the amounts of QRC and RSV extracted with the organic solvents resulted in significantly higher amounts (about 2–3 times) than those in PEG200 and propylene glycol. Although the aim of this work was not the extraction with conventional organic solvents, this evidence could be relevant as it further demonstrated that the bentonite is a valuable source of polyphenols and that the extraction with green solvents might be further improved.

### 3.2. Evaluation of the Antioxidant Power of the Extracts with a DPPH Assay

**Figure 1:**
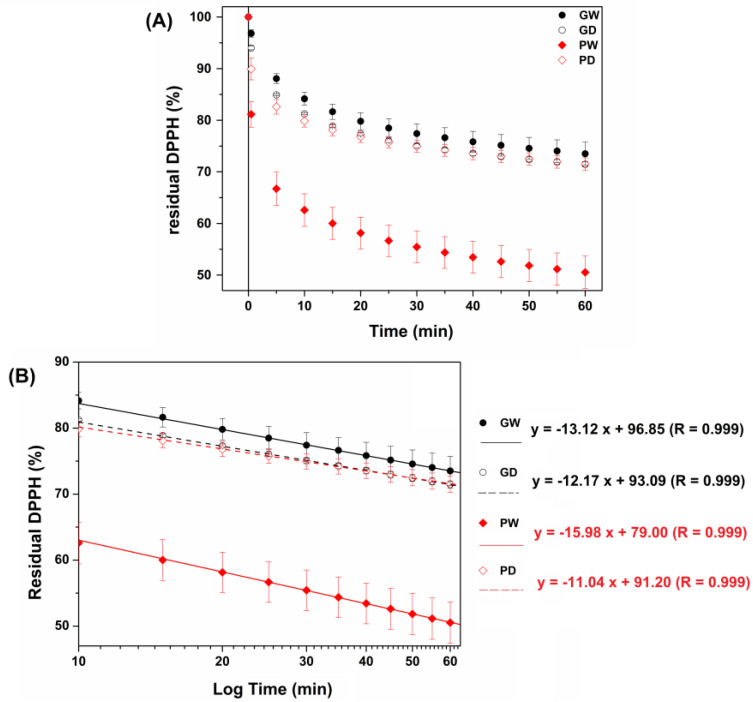
(**A**) Residual DPPH (%) as a function of incubation time when evaluating the GW (black—solid symbol), GD (black—open symbol), PW (red—solid symbol) and PD (red—open symbol) extracts. (**B**) Data in the semi-logarithmic scale and linear curve fitting (linearity range: 20–60 min) (*n* = 9).

To obtain a functional excipient useful in the pharmaceutic and cosmetic fields, the assessment of the antioxidant power of the prepared extracts is a key point. The DPPH assay was thus chosen to compare the antioxidant behavior of the four extracts. It is a simple, well-known and widely used technique, based on the chemical reactivity of the DPPH radical (DPPH^•^) [48,49,50]. The literature reports several DPPH protocols in terms of both DPPH:antioxidant ratio, employed solvent and timing. These crucial parameters were thus accurately evaluated. First, methanol and ethanol were considered solvents for the DPPH assay. Among them, methanol was selected as the methanolic dilution of all the prepared extracts allowed to clear and stable solutions. To identify the best DPPH:extract ratio, several attempts were conducted and, finally, the best results were obtained when diluting 2 mL of DPPH stock solution (40 µg/mL) with 100 µL of each methanolic extract solution (20 µL mL^−1^). Indeed, by increasing the sample amount, the DPPH reduction was too fast while a sample reduction resulted in an excessive slowing down of the DPPH consumption rate; in both cases, any eventual differences were difficult to identify. Additionally, to better compare the antioxidant behavior of the proposed extracts, the kinetic of the DPPH reduction was evaluated instead of considering only one selected time point. The obtained experimental curves are reported in Figure 1A. As observable, all the extracts displayed a starting rapid DPPH consumption for 10 min, followed by a slower but constant consumption rate until the chosen end point (60 min). The latter is much more evident when observing the graph in the semi-logarithmic scale (Figure 1B); indeed, by curve fitting, the experimental points between 20 and 60 min great linear curve-fitting were obtained.

By comparing the tested samples, it is noticeable that the two propylene glycol-based extracts (GW and GD) resulted in quite analogous products, and they were also superimposable with the PD sample (≈70% of residual DPPH after 60 min incubation). Conversely, the PW extract exhibited the highest antioxidant power with a significant and rapid initial DPPH consumption resulting in a ≈50% of residual DPPH at the end point. The slope of the linear plots obtained allows us to appreciate the rate at which the tested extracts exert their antioxidant activity. A larger slope corresponds with a quicker consumption rate of the DPPH reagent. Clearly the PW sample is a quicker antioxidant agent (angular coefficient of 15.98).

To better understand the antioxidant activity of novel investigated extracts, they should be compared to a standard antioxidant molecule. Whereas ascorbic acid is usually the main chosen antioxidant reference, here, gallic acid (GA) was chosen in order to compare potentially similar behaviors: pool of extracted polyphenols versus standard phenolic compound. Then, five standard GA solutions were subjected to the DPPH assay (see Supplementary Materials; Figure S1A,B).

The standard GA solutions all displayed a starting rapid DPPH consumption followed by a plateau. Possibly, the presence of several polyphenols in the extracts allowed the different behaviors characterized by a slow but constant DPPH consumption due to several kinetics of reactions between the antioxidants and the radicals. To better compare the antioxidant power of the extracts with that of the chosen reference, the residual DPPH % at 10, 30 and 60 min was plotted versus the GA solutions concentration thus obtaining three calibration curves. As consequence, knowing the residual DPPH at the selected time points for each extract (sample dilution: 20 µL mL^−1^) and its density, it was possible to express the antioxidant power of 1 g of each extract as equivalent mg of GA (Table 3).

As reported, each sample displayed an equivalent GA concentration, which resulted in an increase by time, confirming that the different antioxidant behaviors respect the GA one.

Once again, PW is the extract with the greater antioxidant power, as 1 g of it possesses an antioxidant power equivalent to 1.322 mg of gallic acid, suggesting that the presence of water in the waste bentonite promotes the extraction of polyphenols when PEG200 was used as a solvent.

### 3.3. Quantification of RSV, GA and QRC with HPLC-DAD Analyses

**Figure 2:**
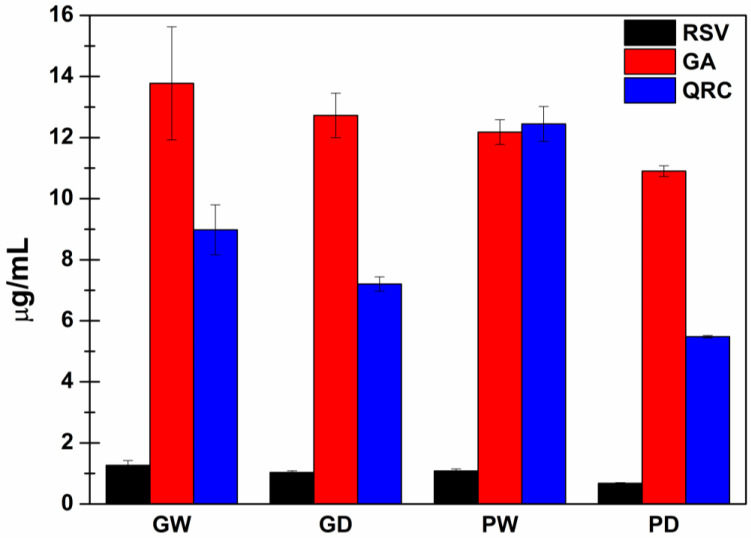
Concentration (μg mL^−1^) ± SE of: Resveratrol (RSV—black), Gallic Acid (GA—red) and Quercetin (QRC—blue) detected in each extract by HPLC-DAD analyses (*n* = 9).

To evaluate the chromatographic profile of the extracted compounds, HPLC-DAD analyses were conducted, and three previously identified phenolic molecules were quantified: Resveratrol (RSV), Gallic Acid (GA) and Quercetin (QRC). The obtained results were expressed as phenols concentration (µg/mL) in each extract and graphically presented in Figure 2.

As observable from both the wet and the dry bentonite samples, high amounts of GA were generally extracted while only small quantities of RSV were recovered. Finally, the QRC concentration into the extracts resulted dependent by the starting presence of water: samples from the wet bentonite displayed a higher QRC concentration than the correspondent extracts from the freeze-dried bentonite (GW > GD and PW > PD). The observed differences were stronger for the PEG200-based samples. In any case, these changes are probably due to enhanced QRC solubility into the selected extraction solvents due to the action of the residual water as effective co-solvent [14,51].

**Figure 3:**
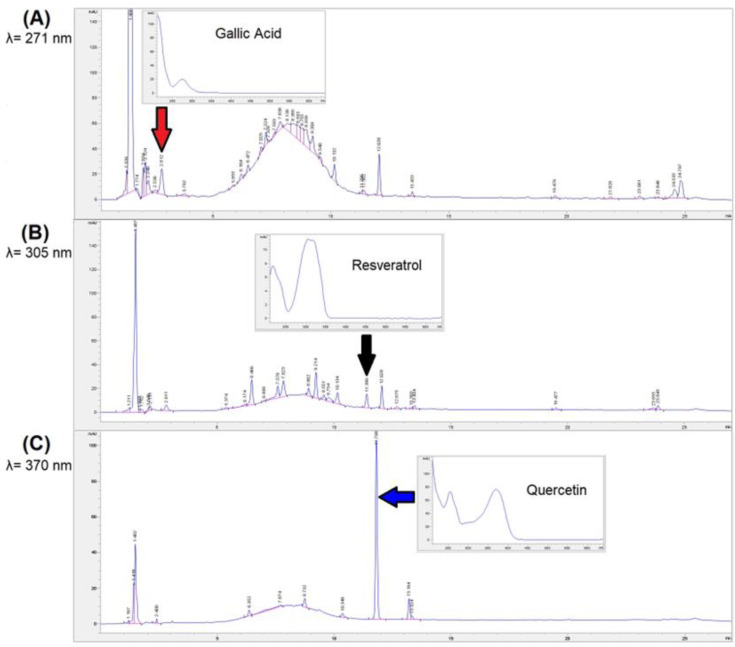
Chromatograms at (**A**) 271 nm; (**B**) 305 nm and (**C**) 370 nm of the PW extract (as a representative sample) employed to evaluate the amount of gallic acid, resveratrol and quercetin respectively. The recorded UV-Vis spectra of the quantified polyphenols are also reported.

Chromatograms at 271, 305 and 370 nm of the PW extract (chosen as a representative sample) are reported in Figure 3 and the 3D plot in Figure S3. The three quantified polyphenols are there highlighted as well as their UV-Vis spectra are reported.

### 3.4. Determination of the Total Phenolic Content with Folin–Ciocalteu Assay

Considering that the HPLC-DAD analyses just allowed to quantify the three previously reported phenolic molecules, the total phenolic content was evaluated with a Folin–Ciocalteu assay to obtain a more complete information regarding the prepared extracts.

**Figure 4:**
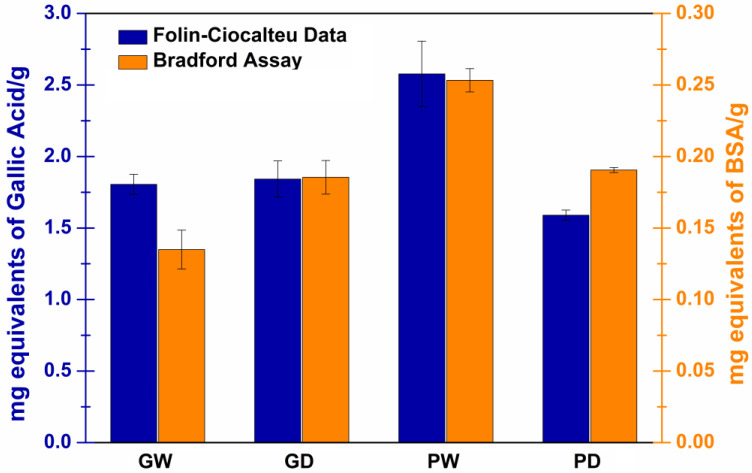
Folin–Ciocalteu data (blue) and Bradford assay results (orange) for all the extracts reported in terms of equivalent mg ± SE of gallic acid and bovine serum albumin used respectively as standard molecules per 1 g of extract (*n* = 9).

The Folin–Ciocalteu assay is a well-known method widely used in clinical and nutritional studies to measure the phenolic content in plant-derived foods and biological samples. It is based on the Folin–Ciocalteu reagent reduction due to reaction with the phenolic compounds in alkaline environment. Clearly, when evaluating a complex sample containing unknown amounts and/or ratios of various polyphenols, it is only possible to evaluate a total phenolic content by referring to a standard molecule. Gallic acid is the most commonly used reference standard, and thus the total phenolic content results are usually expressed as gallic acid equivalent [49,52]. In Figure 4 (blue columns), the obtained results are reported as total phenolic content (in terms of equivalents GA mg) per 1 g of each extract. The reported findings are totally in accordance with the previously reported DPPH results. Indeed, the antioxidant power of the extracts is clearly correlated to the amount of phenolic antioxidant compounds. Thus, the GW, GD and PD samples, which exhibited analogous DPPH reduction patterns, also displayed a comparable total phenolic content. Additionally, in agreement with its greater antioxidant power, the PW sample presented the highest amounts of phenolic molecules.

### 3.5. Determination of the Total Protein Content with a Bradford Assay

The must/wine fining process is mainly aimed at removing proteins and enzymes which otherwise can cause oxidation, turbidity and presence of solid residues in the finished products. Consequently, it is likely to point out that the waste bentonite should contain large amounts of proteins which could be extracted together with the desired functional polyphenols. Indeed, the evaluation of the total protein content of the obtained extracts is needed. The literature reports several methods to determine the total protein content in liquid samples. In this work, the Bradford assay was employed as it is a quick and fairly sensitive method that allows the detection of protein content ranging from 0.2 to 20 μg/mL [53,54]. Again, as an unknown amount and/or ratio of several proteins could be extracted from the starting matrix, it is necessary to refer to a standard protein. Generally, immunoglobulins or bovine serum albumin (BSA) are used reference standard, and consequently, the total protein content of each extract was expressed as equivalent BSA mg per 1 g of each extract (Figure 4, orange columns).

The extracted amount of protein resulted very low in any case (maximum ≈ 0.25 mg per 1 g of PW). Particularly, the GD and PD samples resulted quite comparable thus leading to hypothesize that protein extraction is independent from the chosen extraction solvents. In contrast, the GW sample displayed the smallest value, while the PW sample exhibited the highest one. This could be attributable to different co-solvent effects of the residual water in the waste starting material.

### 3.6. Evaluation of Extracts Safety with a Cytotoxicity Assay

**Figure 5:**
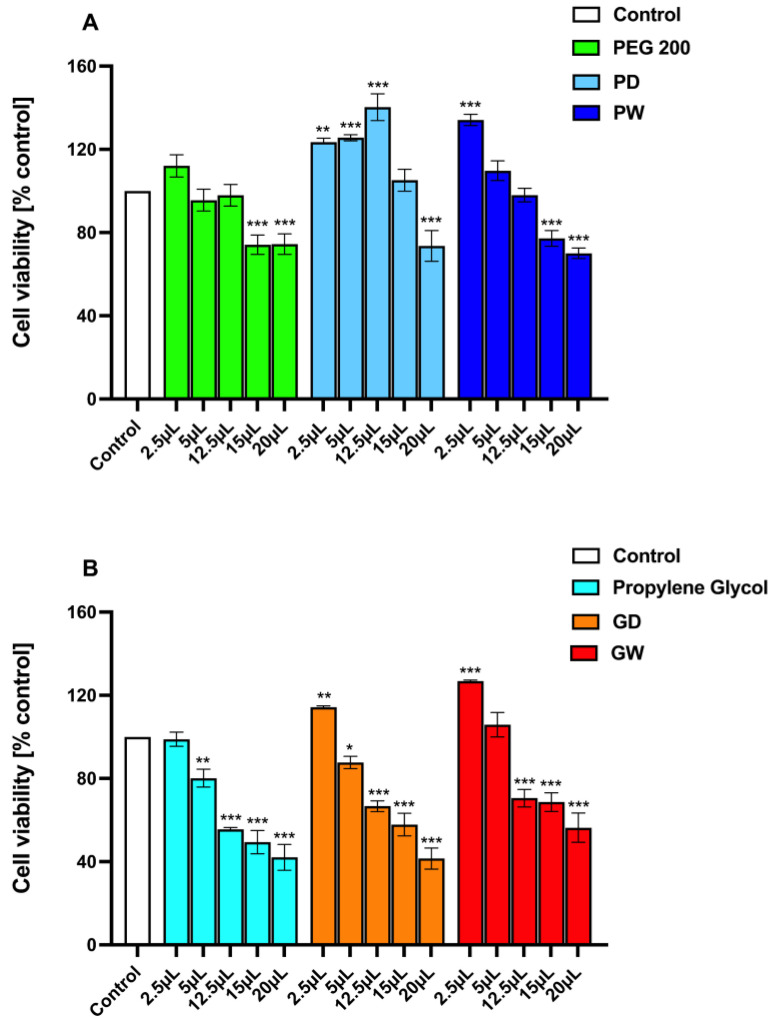
Cell viability assay on BALB/3-T3 cells. Cells were treated for 24 h in the absence (control) or the presence of (**A**) PEG200 and PEG-based extracts, (**B**) Propylene Glycol and Glycol-based extracts at different concentrations (2.5–20 µL/mL). Values are means ±SD of three separate experiments conducted in triplicate. With respect to control, * *p* < 0.05; ** *p* < 0.01; *** *p* < 0.001 (ANOVA associated with Tukey’s test).

Assessing the safety of polyphenols-enriched functional excipients to be directly employed in cosmetics and pharmaceuticals is a crucial aspect to be evaluated. Along these lines, the cytotoxicity of both extracts and extraction solvent were then determined by BALB/3-T3 cells. The tested concentrations (*v*/*v*) were chosen according to the literature [55,56], and the results are reported in Figure 5.

Some relevant considerations could be made by observing the experimental results:

- The fresh extraction solvents under the employed experimental conditions could result in cytotoxic (cell viability % <80%) products in a concentration-dependent manner. This is much more evident when observing the propylene glycol behavior (Figure 5B). However, it should be considered that both PEG200 and propylene glycol are well-known and extensively used cosmetic and pharmaceutic excipients [43,57].
- Generally, all the prepared extracts, at any concentration tested, resulted in less cytotoxic products than the related fresh extraction solvent.
- The extracts displayed a dual behavior. At the lower concentrations tested, they exert proliferative effects while at the higher concentrations evaluated, they caused a decrease in cell viability. These effects might well be attributable to the dual action of the extracted functional polyphenols. Indeed, the literature fully reports that polyphenols could exhibit either proliferative and wound healing or anti-proliferative and anticancer effects depending on their concentration [58,59,60,61,62,63,64].
- The glycol-based extracts (Figure 5B) resulted in a cytotoxic behavior even at low concentrations (≥12.5 µL mL^−1^). Additionally, the GD and GW samples gave comparable results.
- The PEG-based extracts (Figure 5A) resulted in a modest cytotoxicity as even the lower observed cell viability % values were around the 80%. Furthermore, the PD sample exerts a proliferative effect higher than the PW extract. This could be attributable to the already reported highly different content in functional polyphenols, resulting in different in vitro performances.

### 3.7. Stability Studies

**Figure 6:**
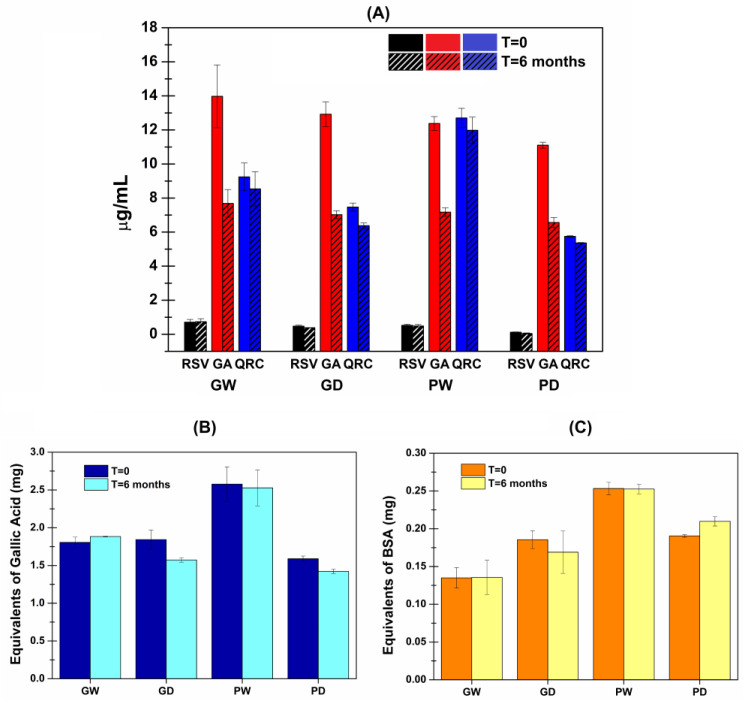
Stability evaluation by comparation of the quantitative analyses conducted on the freshly prepared extract and 6-months-old extracts: (**A**) HPLC-DAD data reported as concentration (μg mL^−1^) ± SE of RSV, QRC and GA; (**B**) Folin–Ciocalteu data reported as equivalent mg ± SE of gallic acid per 1 g of extract; (**C**) Bradford assay results reported as equivalent mg ± SE of BSA per 1 g of extract (*n* = 6).

The last relevant factors evaluated were related to the extracts’ stability because of the well-known instability of polyphenols. Each sample was then stored at 4 °C in the dark for 6 months and subsequently, the stability was assessed both by visual appearance as well as in terms of changed of the previously investigated features (antioxidant power, GA, RSV and QRC amounts by HPLC-DAD, total phenolic and protein content). At the mentioned time point, the extracts resulted in unvaried states in terms of aspect (color, clarity, absence of solid residues). Furthermore, the comparison between the freshly prepared and the 6-month-old extracts is reported in terms of antioxidant power in Table 4 (and also graphically presented as Supplementary Materials: Figure S2) and by the quantitative assays in Figure 6A (HPLC-DAD), B (Folin–Ciocalteu) and C (Bradford assay).

As reported, generally only small changes occurred. The main relevant variations were those related to the GA concentration obtained with the HPLC-DAD analyses. Nevertheless, the obtained results suggest that the extracted pool of polyphenols remained almost stable in the chosen storage conditions thus maintaining its effectiveness as antioxidant. Additionally, the small amounts of extracted proteins also remain stable, thus concurring to not alter the aspect of the extracts over time.

## 4. Conclusions

The waste-to-market approach perfectly fit with the ever-growing perspective of circular economy. In this view a previously considered waste product could become a precious source to be further used to produce novel raw material responding to special market needs. In this paper, some significant findings were highlighted. First, the bentonite from the fining process of must and wine was here promoted from waste to be eliminated to a valuable source of functional polyphenols. Second, a green extraction procedure was optimized by the employment of well-known and widely used cosmetic/pharmaceutical excipients as solvents, an easy and scalable method, low costs and a short time process. In particular, Propylene Glycol and PEG200 were tested as extraction solvents while both wet and freeze-dried waste bentonites were used as extraction matrices. Among the four obtained extracts, the one resulting from the extraction from wet bentonite with PEG200 as a solvent (sample name: PW) emerged as the most promising. It displayed the highest antioxidant power, resulting in a residual DPPH % at 60 min ≈ 50%, making 20 μL of extract powerful as ≈ 30 µg of standard GA while also highlighting a constant antioxidant trend instead of just a rapid starting radicals consumption followed by a plateau. The DPPH data was further confirmed by the HPLC-DAD and Folin–Ciocalteu analyses, which showed the highest amounts of functional polyphenols in the PW sample. These issues remained almost stable after 6-months of extracts storage at 4 °C in the dark. Additionally, PEG200 emerged as more cytocompatible than Propylene Glycol and also PEG-based extracts demonstrated a proliferative behavior. These findings are fully satisfactory and confirm the possibility of considering the waste bentonite a precious novel starting material for polyphenols extraction, as well as the chance of obtaining polyphenols-enriched raw materials with antioxidant power to be further directly used for cosmetic and pharmaceutical products.

## Figures and Tables

**Table 1:** Formula code of the prepared extracts from freeze dried and wet waste bentonite by Propylene Glycol (G) and PEG200 (P).

Formula Code	Bentonite	Solvent
**GW**	2 g of wet bentonite	Propylene Glycol
**GD**	1 g of freeze-dried bentonite	Propylene Glycol
**PW**	2 g of wet bentonite	PEG200
**PD**	1 g of freeze-dried bentonite	PEG200

**Table 2:** Characteristics of the extracts in terms of density, pH and *Yield* % of the extraction process correlated to the respective pure solvents (propylene glycol and PEG200) used as references (*n* = 9).

Formula Code	Density (g/mL)	pH	*Yield* %
**Propylene Glycol**	1.038 ± 0.004	6.66	-
**G_CONTROL_**	1.035 ± 0.005	6.36	-
**GW**	1.053 ± 0.005	4.26	82.85 ± 4.88
**GD**	1.087 ± 0.008	4.32	61.56 ± 6.69
**PEG200**	1.115 ± 0.005	6.39	-
**P_CONTROL_**	1.111 ± 0.007	6.92	-
**PW**	1.134 ± 0.006	4.23	69.32 ± 6.32
**PD**	1.180 ± 0.009	4.44	66.31 ± 2.30

**Table 3:** Antioxidant power of the extracts expressed as equivalent GA mg ± SE at 10, 30 and 60 min per 1 g of extract (*n* = 9).

Sample	Equivalents mg of GA per 1 g of Extract		
	10 min	30 min	60 min
**GW**	0.263 ± 0.051	0.512 ± 0.069	0.608 ± 0.079
**GD**	0.375 ± 0.006	0.579 ± 0.014	0.659 ± 0.018
**PW**	1.073 ± 0.121	1.225 ± 0.105	1.322 ± 0.104
**PD**	0.396 ± 0.043	0.539 ± 0.037	0.606 ± 0.039

**Table 4:** Antioxidant power of the extracts after 6 months storage at 4 °C in the dark, expressed as equivalent GA mg ± SE at 10, 30 and 60 min per 1 g of extract (*n* = 9).

Sample	Equivalents mg of GA per 1 g of Extract		
	10 min	30 min	60 min
**GW**	0.431 ± 0.032	0.627 ± 0.013	0.707 ± 0.009
**GD**	0.361 ± 0.005	0.565 ± 0.001	0.666 ± 0.008
**PW**	0.739 ± 0.034	1.034 ± 0.023	1.178 ± 0.025
**PD**	0.345 ± 0.020	0.523 ± 0.026	0.589 ± 0.027

## Data Availability

Data is contained within the article and supplementary material.

## Supplementary Materials

The following supporting information can be downloaded at: https://www.mdpi.com/article/10.3390/antiox11122493/s1, Figure S1. (A) Residual DPPH (%) as a function of incubation time when evaluating standard GA solutions at concentrations of: 0.050 mg mL^−1^ (black), 0.035 mg mL^−1^ (red), 0.025 mg mL^−1^ (blue), 0.020 mg mL^−1^ (pink) and 0.015 mg m^−1^ (green). (B) Data in the semi-logarithmic scale and linear curve fitting (linearity range: 20–60 min) (*n* = 3); Figure S2. Comparative antioxidant behaviors of the freshly prepared (black and red) and 6-months-old (green) extracts: residual DPPH (%) as a function of incubation time when evaluating the (A) GW, (B) GD, (C) PW and (D) PD extracts (*n* = 6). Figure S3. 3D plot image of the PW extract: elution from 0 to 27 min.
