# Supplementary material for: Green Extraction of Polyphenols from Waste Bentonite to Produce Functional Antioxidant Excipients for Cosmetic and Pharmaceutical Purposes: A Waste-to-Market Approach
Source: Antioxidants (Basel). 2022 Dec 19;11(12):2493. doi: 10.3390/antiox11122493 (PMC9774313; doi:10.3390/antiox11122493)
Supplement: Supplementary file 1 [file antioxidants-11-02493-s001.zip › antioxidants-2077273-supplementary.pdf]

Supplementary Materials

Article

Green extraction of polyphenols from waste bentonite to produce functional antioxidant excipients for cosmetic and pharmaceutical purposes: a waste-to-market approach

Giulia Di Prima[1], Elena Belfiore[2], Martina Migliore[1], Amalia Giulia Scarpaci[1], Giuseppe Angellotti[2], Ignazio Restivo[1], Mario Allegra[1], Vincenzo Arizza[1], and Viviana De Caro [1,3]*

Data reported as supplementary material:

- **Figure S1:** A) Residual DPPH (%) as a function of incubation time when evaluating standard GA solutions at concentrations of: 0.050 mg mL^{-1} (black), 0.035 mg mL^{-1} (red), 0.025 mg mL^{-1} (blue), 0.020 mg mL^{-1} (pink) and 0.015 mg mL^{-1} (green). B) Data in the semi-logarithmic scale and linear curve fitting (linearity range: 20-60 min) (n=3)
- **Figure S2:** Comparative antioxidant behaviors of the freshly prepared (black and red) and 6-months-old (green) extracts: residual DPPH (%) as a function of incubation time when evaluating the A) GW, B) GD, C) PW and D) PD extracts (n=6)
- **Figure S3:** 3D plot image of the PW extract: elution from 0 to 27 min.

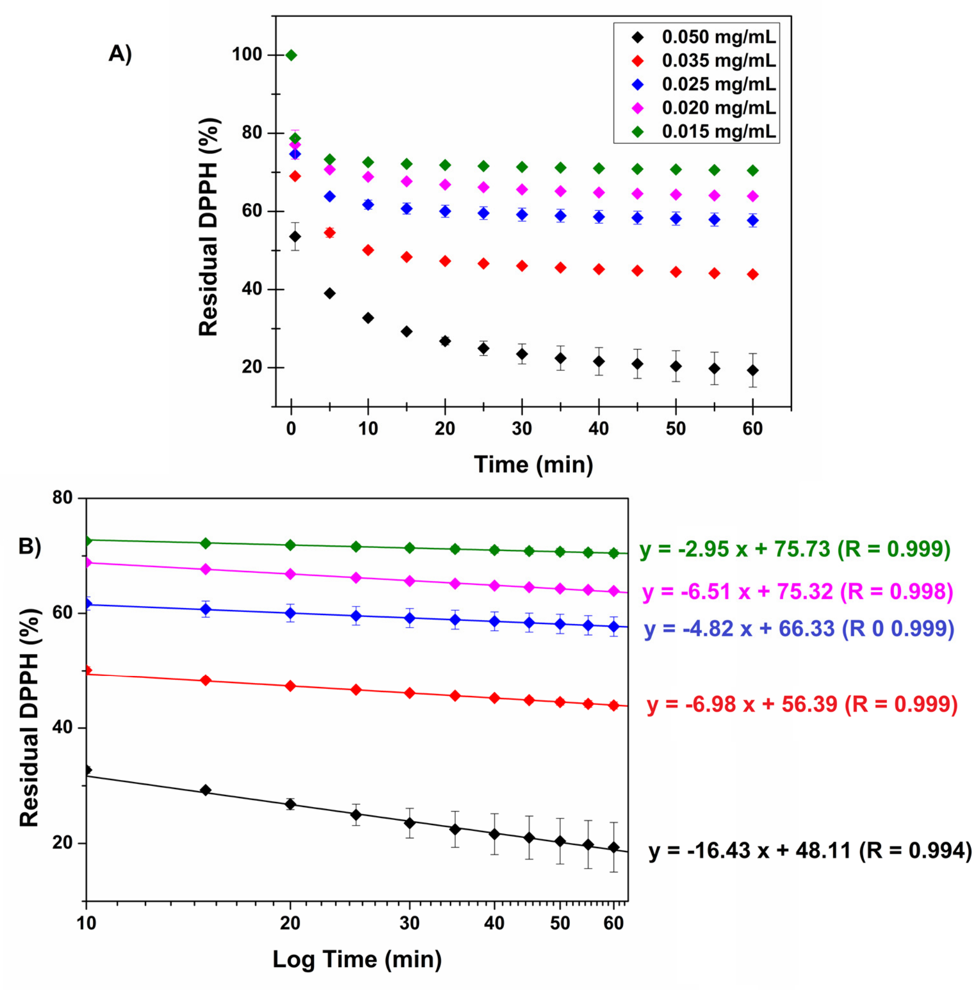

Figure S1. A) Residual DPPH (%) as a function of incubation time when evaluating standard GA solutions at concentrations of: 0.050 mg mL^{-1} (black), 0.035 mg mL^{-1} (red), 0.025 mg mL^{-1} (blue), 0.020 mg mL^{-1} (pink) and 0.015 mg mL^{-1} (green). B) Data in the semi-logarithmic scale and linear curve fitting (linearity range: 20-60 min) (n=3)

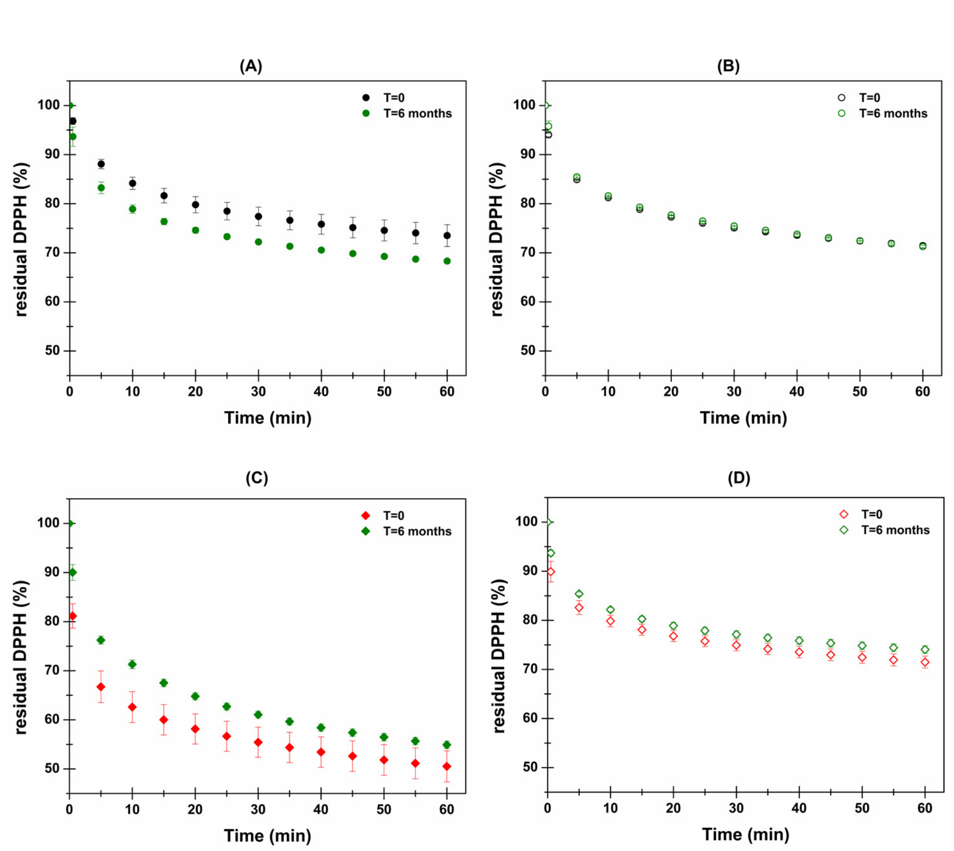

Figure S2. Comparative antioxidant behaviors of the freshly prepared (black and red) and 6-months-old (green) extracts: residual DPPH (%) as a function of incubation time when evaluating the A) GW, B) GD, C) PW and D) PD extracts (n=6)

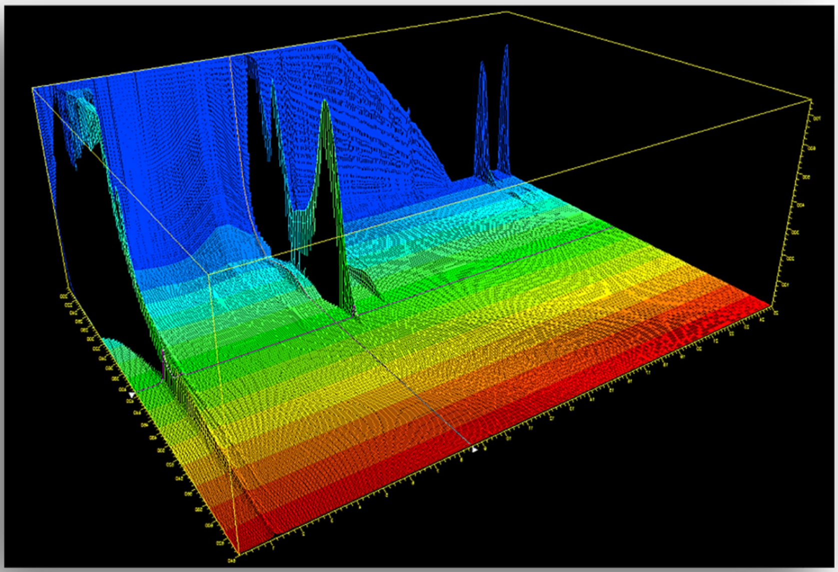

Figure S3: 3D image of PW extract elution from 0 to 27 min.
